# The TaCOMT1A‐Synthesised Sakuranetin Is a Novel Biostimulant Enhancing Drought Tolerance and Yield in Wheat

**DOI:** 10.1111/pbi.70734

**Published:** 2026-08-07

**Authors:** Mingzhao Luo, Chengjie Xu, Wenjing Yang, Wensi Tang, Kai Chen, Jiaqing Guo, Qiyu Wang, Jun Chen, Zhaoshi Xu, Pierre Delaplace, Youzhi Ma, Yongbin Zhou, Ming Chen

**Affiliations:** ^1^ State Key Laboratory of Crop Gene Resources and Breeding, Key Laboratory of Biology and Genetic Improvement of Triticeae Crops, Ministry of Agriculture, Institute of Crop Sciences Chinese Academy of Agricultural Sciences (CAAS) Beijing China; ^2^ Tianjin Crop Research Institute Tianjin Academy of Agricultural Sciences Tianjin China; ^3^ Plant Genetics and Rhizosphere Processes Laboratory, Gembloux Agro‐Bio Tech, Teaching and Research Centre (TERRA), University of Liège Gembloux Belgium; ^4^ Key Laboratory of Sustainable Dryland Agriculture, College of Agriculture Shanxi Agricultural University Jinzhong China; ^5^ Key Laboratory of Grain Crop Genetic Resources Evaluation and Utilisation, Institute of Crop Sciences Chinese Academy of Agricultural Sciences Beijing China

**Keywords:** caffeic acid O‐methyltransferase, drought tolerance, gibberellic acid, plant height, sakuranetin, wheat

## Abstract

Drought stress is a major limitation to global wheat production. Here, we demonstrate that the wheat gene *TaCOMT1A*, encoding a caffeic acid O‐methyltransferase, plays a crucial role in enhancing drought tolerance in wheat. Overexpression of *TaCOMT1A* significantly improved drought tolerance at the seedling stage, as evidenced by higher survival rates, biomass, and antioxidant capacity, along with reduced oxidative damage in transgenic lines. Field trials demonstrated that these lines maintained superior grain yield under limited irrigation. We identified TaCOMT1A as a multifunctional enzyme capable of synthesising both the flavonoid sakuranetin and melatonin in vitro. Metabolomic and functional analyses confirmed that sakuranetin is a key downstream metabolite mediating the drought tolerance conferred by *TaCOMT1A*. Exogenous application of sakuranetin enhanced drought tolerance across diverse wheat cultivars and, importantly, rescued the susceptible phenotype of *TaCOMT1A* EMS mutants (E829 and E830). Mechanistically, sakuranetin treatment bolstered the antioxidant system and attenuated oxidative stress under drought. Furthermore, both *TaCOMT1A* overexpression and sakuranetin application reduced plant height by suppressing gibberellic acid (GA_3_) biosynthesis. Crucially, field application of sakuranetin increased grain yield under both well‐irrigated and drought conditions. Our results establish a novel pathway where *TaCOMT1A* enhances drought tolerance and modulates plant architecture primarily through the production of sakuranetin, positioning this metabolite as a promising plant‐based priming agent for sustainable wheat cultivation.

## 1 Introduction

Rising global population and the increasing frequency of extreme climatic events, such as drought, pose a significant threat to sustainable food production (Del Buono 2021). While developing resilient crop cultivars is essential, complementary strategies utilising agrochemicals to enhance intrinsic plant tolerance are critical. Synthetic plant growth regulators (PGRs), such as the gibberellin biosynthesis inhibitor paclobutrazol, can ameliorate drought stress and improve yield (Ma et al. 2022). However, their potential environmental and non‐target effects necessitate precise application and underscore the need for safer alternatives (Chen et al. 2020). Plant‐derived biostimulants represent a promising alternative, leveraging natural compounds to enhance stress resilience and nutrient use efficiency with a reduced ecological footprint (Johnson et al. 2024).

Flavonoids, a major class of plant secondary metabolites, are prime candidates for biostimulant development due to their central role in stress adaptation, primarily through reactive oxygen species (ROS) scavenging (Jogawat et al. 2021). Among these, the flavanone phytoalexin sakuranetin is a key defence compound in rice, conferring resistance against pathogens and herbivores (Liu et al. 2023; Shimizu et al. 2012). Its involvement in abiotic stress responses suggests a broader protective function, yet its efficacy and mechanism as an applied biostimulant in crops like wheat remain unexplored.

Biosynthesis of such protective flavonoids is often governed by enzymes within the phenylpropanoid pathway (Lam et al. 2023). Caffeic acid O‐methyltransferase (COMT) is a multifunctional enzyme critical for synthesising diverse metabolites, including lignin, flavonoids, and melatonin (Shen et al. 2022). By modulating these pathways, COMT influences agronomically vital traits such as lodging resistance, stress tolerance, and yield (Huangfu et al. 2022). We previously established that the wheat gene *TaCOMT1A* improves drought tolerance in Arabidopsis via enhanced melatonin synthesis (Yang et al. 2019). However, its function in wheat, its potential role in flavonoid metabolism, and its downstream mechanisms are unknown.

We hypothesised that *TaCOMT1A* contributes to drought resilience in wheat by catalysing the production of protective metabolites, potentially including sakuranetin, and that this flavonoid itself could function as an effective biostimulant. This study aimed to: (1) delineate the role of *TaCOMT1A* in wheat drought tolerance under controlled and field conditions, (2) elucidate its biochemical function in relation to sakuranetin, and (3) evaluate the biostimulant potential and molecular mechanism of exogenous sakuranetin application. Our results identify sakuranetin as a novel, dual‐action agent that simultaneously enhances drought tolerance and reduces plant height, offering a strategic tool to improve wheat productivity and lodging resistance.

## 2 Results

### 2.1 Exogenous Sakuranetin Improves Drought Tolerance and Grain Yield of Wheat Under Field Conditions

Sakuranetin, a plant‐derived flavonoid with documented biotic stress resistance, has emerged as a potential candidate for abiotic stress management in crops. To validate its practical utility in wheat production, we conducted multi‐location field trials to evaluate the agronomic performance of drought‐sensitive cultivar ‘Shi4185’ following exogenous sakuranetin application.

**FIGURE 1:**
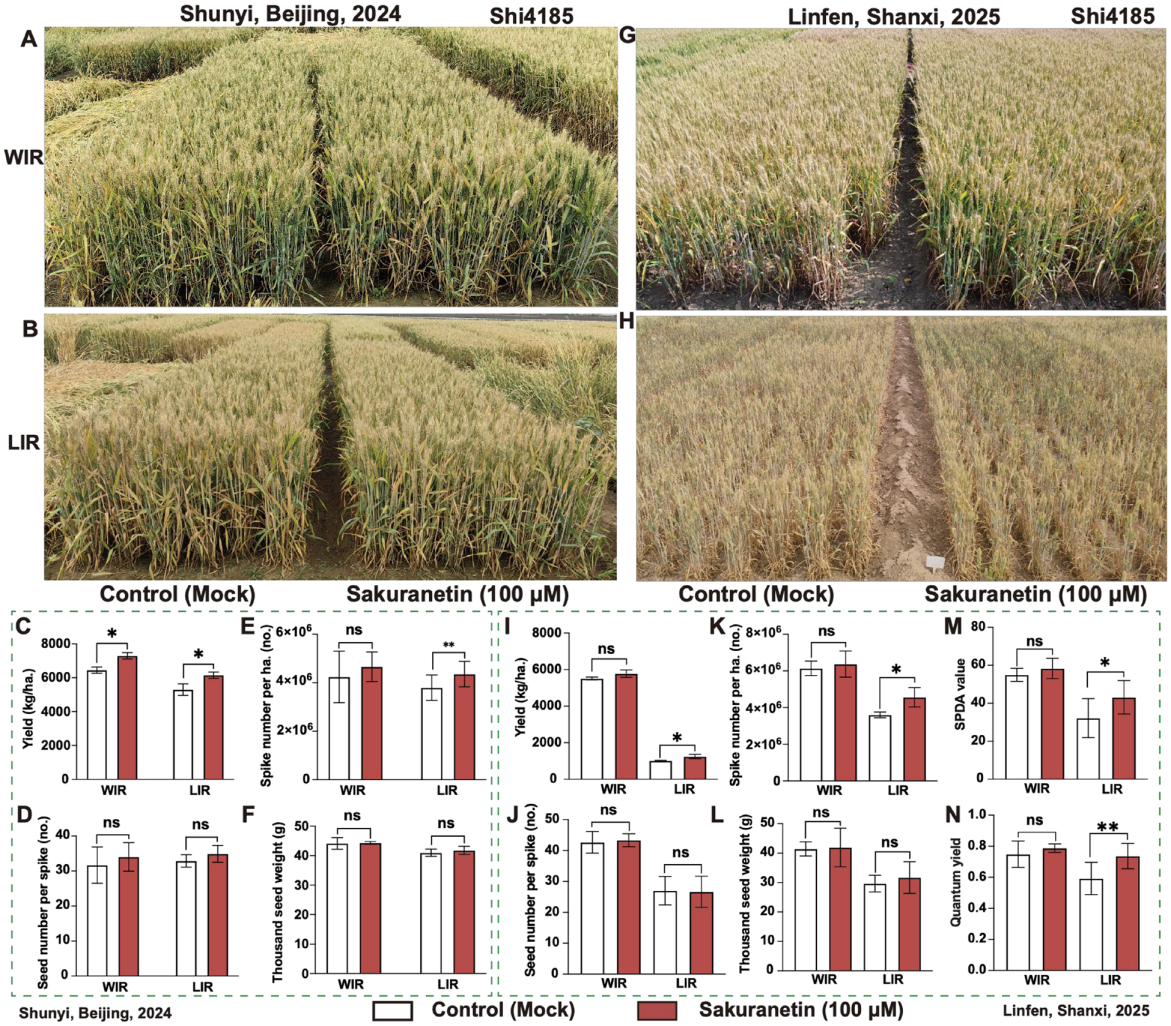
Field performance and agronomic traits of wheat cultivar ‘Shi4185’ in response to exogenous sakuranetin. (A, B) Phenotype of ‘Shi4185’ at the grain‐filling stage under (A) well‐irrigated (WIR) and (B) limited irrigation (LIR, drought stress) conditions after treatment with water (control) or 100 μmol sakuranetin in Shunyi, Beijing, 2024. (C–F) Quantification of key agronomic traits at maturity: (C) grain yield per hectare, (D) seed number per spike, (E) spike number per hectare, (F) thousand‐seed weight. (G, H) Phenotype of ‘Shi4185’ at the grain‐filling stage under (G) well‐irrigated (WIR) and (H) limited irrigation (LIR, drought stress) conditions after treatment with water (control) or 100 μmol sakuranetin in Linfen, Shanxi, 2025. (I–L) Quantification of key agronomic traits at maturity: (I) grain yield per hectare, (J) seed number per spike, (K) spike number per hectare, (L) thousand‐seed weight. (M) SPDA value and quantum yield (N) under WIR and LIR conditions. All bar graphs show mean ± SD (*n* = 3 independent field plot replicates). Significant differences between sakuranetin‐treated and control plants within the same irrigation regime were determined by Student's *t*‐test (*p < 0.05, **p < 0.01). ns, not significant.

Sakuranetin solution (100 μM) was sprayed at a volume of 450 L/ha at the wheat jointing stage, with two applications per experimental plot (see Materials and Methods). Under both well‐irrigated (WIR) and limited irrigation (LIR, drought stress) conditions, sakuranetin treatment significantly improved plant performance and yield‐related traits (Figure 1). Quantitative analysis showed that grain yield per plant was increased by 13.22% under WIR and 16.08% under LIR in the 2024 field trial (Shunyi, Beijing, China) (Figure 1A–F, Table S2). In the semi‐arid region of Linfen, Shanxi, China (2025), sakuranetin application enhanced the yield of ‘Shi4185’ by 18.6% under drought stress (Figure 1G–N, Table S3). These results confirm that exogenous sakuranetin effectively enhances drought resilience and stabilises grain yield across diverse environments.

### 2.2 Overexpression of *TaCOMT1A* Enhances Field Drought Resistance, Yield Performance and Lodging Tolerance

Our previous study demonstrated that wheat *TaCOMT1A* improves drought tolerance in transgenic *Arabidopsis* by regulating melatonin biosynthesis (Yang et al. 2019), and its transcript levels are significantly upregulated in wheat roots and leaves under drought stress. To further characterise its function in wheat, we generated eight homozygous T_3_ transgenic lines overexpressing *TaCOMT1A* (OE lines). Molecular identification confirmed successful transgene integration (Figure S1B) and significantly higher *TaCOMT1A* expression in all OE lines compared to wild‐type (WT) ‘Fielder’, with lines #6, #8, and #9 showing the highest transcript abundance (Figure S1C). These three lines were selected for subsequent functional assays.

**FIGURE 2:**
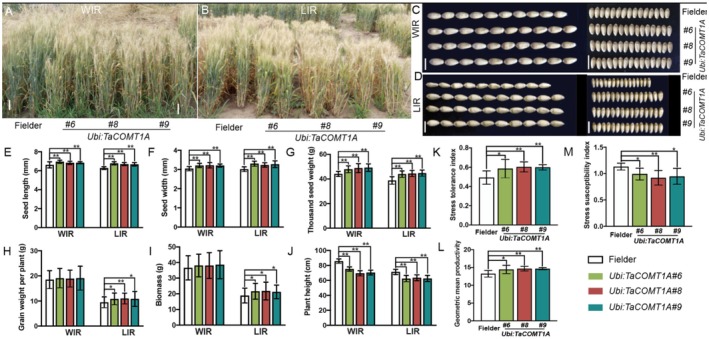
*TaCOMT1A* overexpression enhances drought tolerance and agronomic performance in field trials in 2022. (A, B) Phenotype of wild‐type ‘Fielder’ and *TaCOMT1A*‐overexpressing (OE) lines under well‐irrigated (WIR) (A) and limited irrigation (LIR, drought stress) (B) conditions at the grain‐filling stage. Scale bar = 10 cm. (C, D) Representative grain morphology from plants grown under WIR (C) and LIR (D) conditions. Scale bar = 1 cm. (E–J) Quantitative agronomic traits under WIR and LIR conditions: Seed length (E), seed width (F), thousand‐seed weight (G), grain weight per plant (H), aboveground biomass (I), and plant height (J). (K–M) Drought tolerance indices: Stress Tolerance Index (STI, K), Geometric Mean Productivity (GMP, L), and Stress Susceptibility Index (SSI, M). A randomised block design with three replicates was used. Sowing density was 50 seeds. LIR plants received two irrigations (pre‐wintering and jointing stages); WIR plants received three irrigations (pre‐wintering, jointing, and filling stages). Each irrigation applied 750 m^3^ ha^−1^. All bar graphs show mean ± SD (*n* = 15 biologically independent plants). Significant differences between each OE line and the wild‐type ‘Fielder’ within the same irrigation regime were determined by Student's *t*‐test (*p < 0.05, **p < 0.01). NS, not significant.

Field trials conducted in Shunyi, Beijing, China (2022) showed that *TaCOMT1A* overexpression altered key agronomic traits and improved drought adaptation (Figure 2). OE lines exhibited a moderate semi‐dwarf phenotype with reduced plant height (Figure 2A,B) and produced larger grains, with significant increases in grain length, grain width, and thousand‐seed weight (Figure 2C–G; Tables S4, S5). Under LIR, grain weight per plant and total biomass of OE lines were markedly higher than those of WT (Figure 2H,I), while a 6.72%–7.39% yield increase was observed under WIR. Consistent with enhanced drought tolerance, OE lines had higher stress tolerance index (STI) and geometric mean productivity (GMP), as well as lower stress susceptibility index (SSI) (Figure 2K–M). A repeated field trial in 2023 yielded consistent results (Figure S2; Tables S6, S7), confirming the stability of the phenotype.

Further analysis of plant architecture revealed that *TaCOMT1A* overexpression shortened internode length (particularly the 1st, 2nd, and 5th internodes), increased stem diameter (2nd, 3rd, and 4th internodes), and elevated stem lignin content (Figure S3B–F). These modifications enhanced stem mechanical strength, resulting in superior lodging resistance under wind stress (Figure S3A) and increased stem puncture resistance (Figures S3G and S5H). Collectively, these data demonstrate that *TaCOMT1A* overexpression simultaneously improves drought tolerance, grain yield, and lodging resistance in field‐grown wheat.

### 2.3 TaCOMT1A Overexpression Enhances Drought Tolerance at the Seedling Stage Under Greenhouse Conditions

To provide auxiliary evidence for the field phenotypes, we evaluated the drought tolerance of *TaCOMT1A*‐OE lines at the seedling stage under greenhouse conditions. Under progressive drought treatment, OE seedlings showed better growth than WT at 15 days after drought (DAD) and following rehydration (Figure S4A–C). After rewatering, the survival rate and shoot fresh weight of OE lines were significantly higher than those of WT (Figure S4D,E), and OE lines maintained higher shoot dry weight under stress (Figure S4F).

Physiologically, drought‐induced accumulation of malondialdehyde (MDA) and hydrogen peroxide (H_2_O_2_) was significantly reduced in OE lines, accompanied by elevated catalase (CAT) activity (Figure S4G–I). NBT and DAB staining further confirmed that *TaCOMT1A* overexpression mitigated drought‐triggered reactive oxygen species (ROS) burst (Figure S5A,B). These results indicate that *TaCOMT1A* positively regulates drought tolerance at both the seedling and mature stages.

### 2.4 TaCOMT1A Acts as a Multifunctional Enzyme Catalysing the Biosynthesis of Sakuranetin and Melatonin

To uncover the metabolic basis of *TaCOMT1A*‐mediated stress tolerance, we performed metabolomic analysis of OE lines and WT. Among 158 detected flavonoids, 55 were significantly decreased, and 5 were increased in OE lines, including sakuranetin, isorhamnetin, and vitexin (Data S1). Exogenous application of these three flavonoids enhanced drought tolerance in wheat seedlings, suggesting their potential role in stress responses.

**FIGURE 3:**
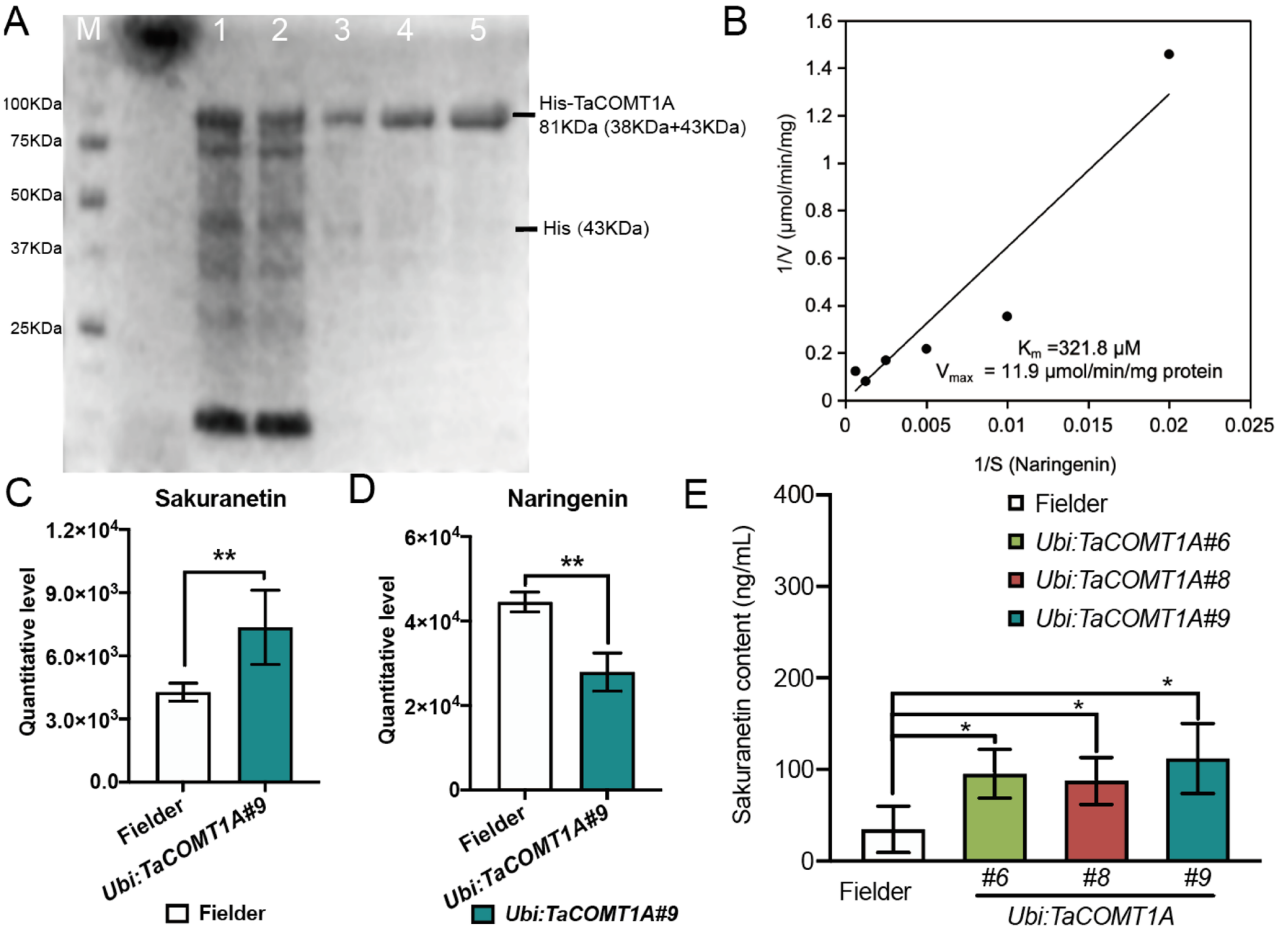
Biochemical characterisation of TaCOMT1A enzyme activity and in vivo sakuranetin production. (A) Purification of recombinant TaCOMT1A. His‐tagged TaCOMT1A was expressed in *Escherichia coli* and purified via nickel‐affinity chromatography. M: Protein molecular weight marker. Lanes 1–2: Total bacterial proteins before (lane 1) and after (lane 2) induction with 0.5 mM isopropyl‐β‐d‐thiogalactoside (IPTG). Lanes 3–5: Eluted, purified His‐TaCOMT1A protein. (B) In vitro enzyme kinetics of TaCOMT1A. Michaelis–Menten plot of TaCOMT1A activity. Purified enzyme (1 μg) was incubated for 60 min at 37°C with varying concentrations of naringenin and 200 μmol S‐adenosylmethionine (SAM). Sakuranetin was quantified by HPLC. Kinetic parameters (K_m_ and V_max_) were derived from nonlinear regression of the data. (C, D) In vivo metabolite levels in wild‐type and *TaCOMT1A*‐overexpressing wheat. (C) Sakuranetin and (D) its biosynthetic precursor naringenin were quantified by UPLC‐MS/MS in the shoots of wild‐type ‘Fielder’ and the homozygous *TaCOMT1A*‐overexpression line #9 under control conditions. (E) Sakuranetin accumulation in independent overexpression lines. Sakuranetin content was measured by HPLC in the shoots of wild‐type ‘Fielder’ and three independent *TaCOMT1A*‐overexpression lines (#6, #8, and #9). Data are presented as mean ± SD (*n* = 3 biologically independent samples). Significant differences between overexpression lines and the wild‐type ‘Fielder’ were determined by Student's *t*‐test (*p < 0.05, **p < 0.01).

In vitro enzyme activity assays validated that recombinant TaCOMT1A protein specifically catalyses the conversion of naringenin to sakuranetin (K_m_ = 321.8 μmol; V_max_ = 11.9 μmol/min/mg), but not isorhamnetin or vitexin (Figure 3A,B). Metabolite profiling confirmed a significant decrease in naringenin (substrate) and a concomitant increase in sakuranetin (product) in OE lines (Figure 3C–E). Additionally, TaCOMT1A catalysed melatonin synthesis from N‐acetylserotonin (NAS) with kinetic parameters K_m_ = 5.02 μmol and V_max_ = 12.06 μmol/min/mg (Figure S6A), and endogenous melatonin content was significantly higher in OE lines (Figure S6B).

Phylogenetic analysis of COMT family proteins from wheat (retrieved from Phytozome, https://phytozome‐next.jgi.doe.gov) and rice (retrieved from the National Rice Data Center, https://www.ricedata.cn/gene/) showed that TaCOMT1A clusters closely with rice COMT homologues rather than rice naringenin 7‐O‐methyltransferase (OsNOMT), the enzyme responsible for sakuranetin biosynthesis in rice (Figure S7). UPLC‐MS/MS chromatograms of standard samples and enzymatic reactions confirmed the specific detection and quantification of sakuranetin and melatonin (Figure S8A–D). Collectively, these results identify TaCOMT1A as a multifunctional O‐methyltransferase mediating the biosynthesis of both sakuranetin and melatonin in wheat.

### 2.5 Exogenous Sakuranetin Application Recapitulates the Drought‐Tolerant Phenotypes of TaCOMT1A Overexpression

**FIGURE 4:**
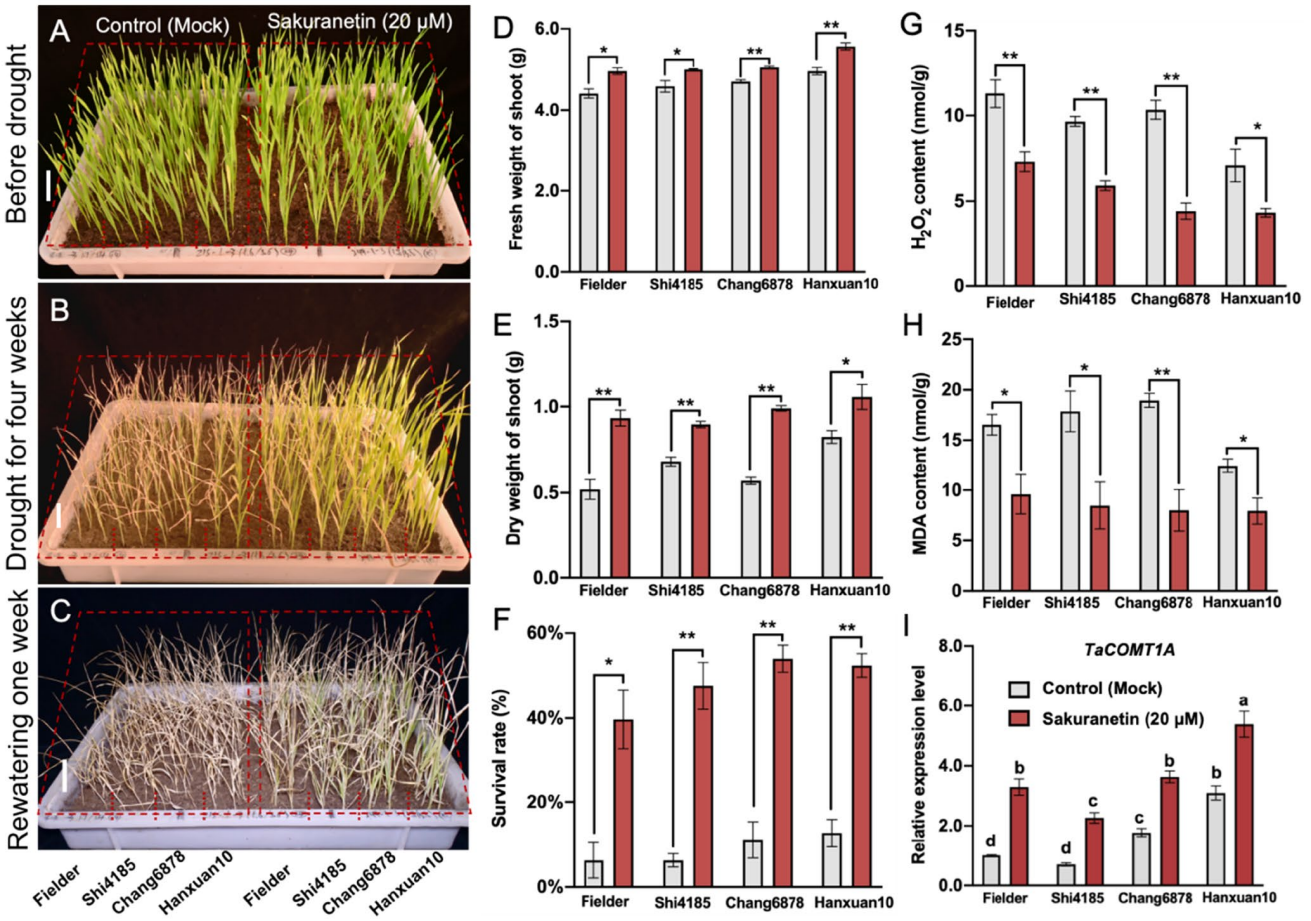
Exogenous application of sakuranetin enhances drought tolerance across multiple conventional wheat cultivars under greenhouse conditions. (A–C) Representative phenotypes of four wheat cultivars (‘Fielder’, ‘Shi 4185’, ‘Chang 6878’, and ‘Hanxuan 10’) following drought treatment. All germinated seeds were planted in Pindstrup peat‐based soil moistened with water at a 1:2 ratio (1 g soil: 2 mL water). Pots were subjected to drought stress beginning at 7 days post‐planting. (A) Phenotype on the first day of drought (1 DAD). (B) Phenotype after four weeks of drought stress. Plants were pre‐treated with five applications of either water (Mock) or 20 μM sakuranetin before the initiation of drought. (C) Phenotype after one week of rehydration following the drought period. (D, E) Shoot fresh weight (D) and dry weight (E) of the four cultivars following three weeks of drought stress with or without sakuranetin pre‐treatment. (F) Survival rates of the four cultivars after rehydration. (G, H) Hydrogen peroxide (H_2_O_2_) content (G) and malondialdehyde (MDA) content (H) in shoots after four weeks of drought stress with or without sakuranetin pre‐treatment. (I) Relative expression level of *TaCOMT1A* in different wheat cultivars (‘Fielder’, ‘Shi 4185’, ‘Chang 6878’, and ‘Hanxuan 10’) under drought and sakuranetin + drought conditions. Data are presented as mean ± SD (*n* = 3 biological replicates, with 6 seedlings per replicate). Asterisks indicate significant differences between sakuranetin‐treated and water‐treated control plants of the same cultivar at the same time point, as determined by Student's *t*‐test (*p < 0.05; **p < 0.01; ns, not significant). Different lowercase letters indicate significant differences among wheat cultivars at the same time point (one‐way ANOVA, Duncan's test, *p < 0.05). Scale bars (in A to C) = 2 cm.

We further explored the biological function of sakuranetin using five wheat cultivars with distinct drought sensitivity. Exogenous application of 10–20 μmol sakuranetin enhanced drought tolerance across all cultivars, as evidenced by higher survival rates, shoot fresh weight, and relative water content under drought (Figure S9A–M). A split‐pot experiment eliminated microenvironmental interference, confirming that sakuranetin‐treated plants (20 μmol) had significantly improved water status compared to mock‐treated controls in the same pot (Figure 4A–F).

Consistent with enhanced drought tolerance, sakuranetin‐treated plants accumulated lower levels of H_2_O_2_ and MDA (Figure 4G,H; Figure S9N,O), indicating strengthened antioxidant defence. Drought‐sensitive cultivar ‘Shi4185’ showed a more pronounced response to sakuranetin, which correlated with its lower basal *TaCOMT1A* expression (≈3.0‐fold lower than drought‐tolerant ‘Hanxuan10’) (Figure 4I).

Genetic verification using EMS‐induced *TaCOMT1A* loss‐of‐function mutants (in the ‘Jing411’ background) showed that mutant seedlings had reduced fresh weight, dry weight, and survival rate under drought. Importantly, exogenous sakuranetin application fully rescued the drought‐sensitive phenotype of these mutants (Figure S10A–L), confirming that sakuranetin is a key downstream mediator of TaCOMT1A function.

### 2.6 Transcriptomic Analysis Reveals Sakuranetin‐Mediated Transcriptional Reprogramming Under Drought

To dissect the molecular mechanisms of sakuranetin‐mediated drought tolerance, we performed RNA‐seq analysis on three wheat cultivars with contrasting drought sensitivity: ‘Shi4185’ (sensitive), ‘Chang6878’ (tolerant), and ‘Hanxuan10’ (tolerant). Seedlings were subjected to four treatments: well‐watered (W), well‐watered + sakuranetin (SW), drought (D), and drought + sakuranetin (SD). Sequencing of 36 samples (3 biological replicates per treatment) generated high‐quality data (≥ 6.88 GB per sample, 95.4%–96.61% mapping rate) (Data S2).

Differentially expressed gene (DEG) analysis (|log1.5FC|> 1, adj. *p* < 0.05) showed that drought stress induced extensive transcriptional reprogramming, with ‘Shi4185’ exhibiting the most DEGs (38732), followed by ‘Chang6878’ (26570) and ‘Hanxuan10’ (26376) (Figure S11A; Data S3–S5). Sakuranetin alone (SW vs. W) triggered fewer DEGs, with tolerant cultivars showing a more robust response. Under combined drought and sakuranetin treatment (SD vs. D), sakuranetin specifically modulated a subset of drought‐responsive genes (Figure S11A). Intersection analysis identified core DEG sets regulated by both sakuranetin and drought, as well as sakuranetin‐specific DEGs under well‐watered conditions (Figure S11B–D).

**FIGURE 5:**
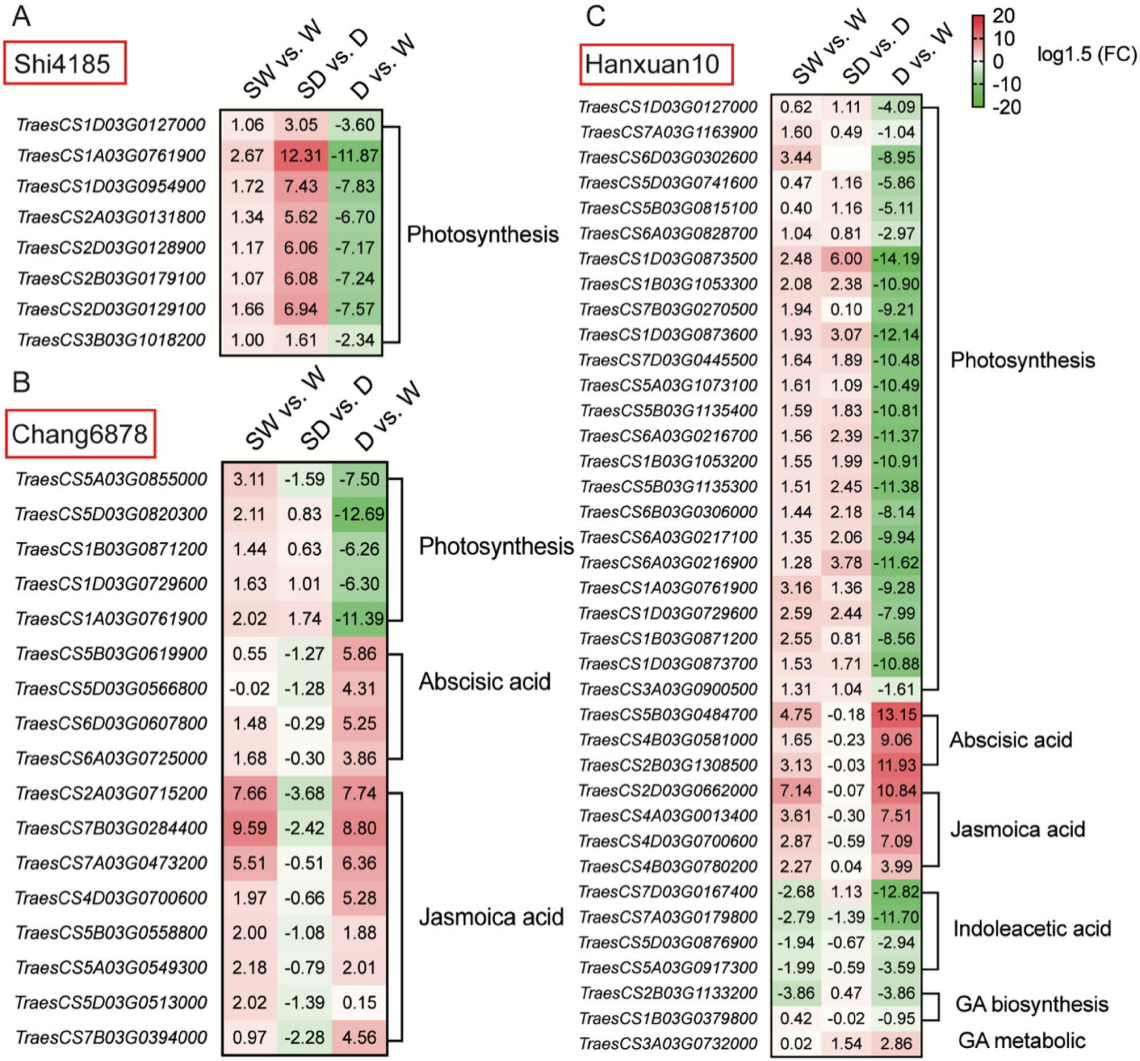
Transcriptional reprogramming of key pathways by sakuranetin under drought stress in wheat cultivars. (A–C) Heatmap showing the expression patterns of differentially expressed genes (DEGs) in major Kyoto Encyclopedia of Genes and Genomes (KEGG) pathways involved in sakuranetin‐mediated drought response in three wheat cultivars: ‘Shi4185’ (A), ‘Chang6878’ (B), and ‘Hanxuan10’ (C). Gene expression levels (log1.5‐transformed FPKM values) are represented by the colour scale from low (green) to high (red). Treatment groups: W (well‐watered control), SW (well‐watered + sakuranetin), D (drought stress), SD (drought stress + sakuranetin). Sample labels: S_ (‘Shi4185’), C_ (‘Chang6878’), H_ (‘Hanxuan10’).

KEGG enrichment analysis revealed that sakuranetin stabilises photosynthetic pathways and modulates hormone signalling under stress (Figure 5A–C, Dataset S6). Genes associated with photosynthesis (e.g., chlorophyll biosynthesis, rubisco, photosystem components) were downregulated by drought but maintained or upregulated by sakuranetin. In tolerant cultivars, sakuranetin dynamically regulated ABA/JA signalling (upregulated under W, downregulated under D) to prime stress responses while avoiding over‐activation. Additionally, sakuranetin enhanced the expression of drought‐related transcription factors (WRKY, MYB, NAC, DREB) under drought (Figure S12A–C).

### 2.7 Sakuranetin Modulates Gibberellin Biosynthesis to Coordinate Drought Tolerance and Plant Growth

**FIGURE 6:**
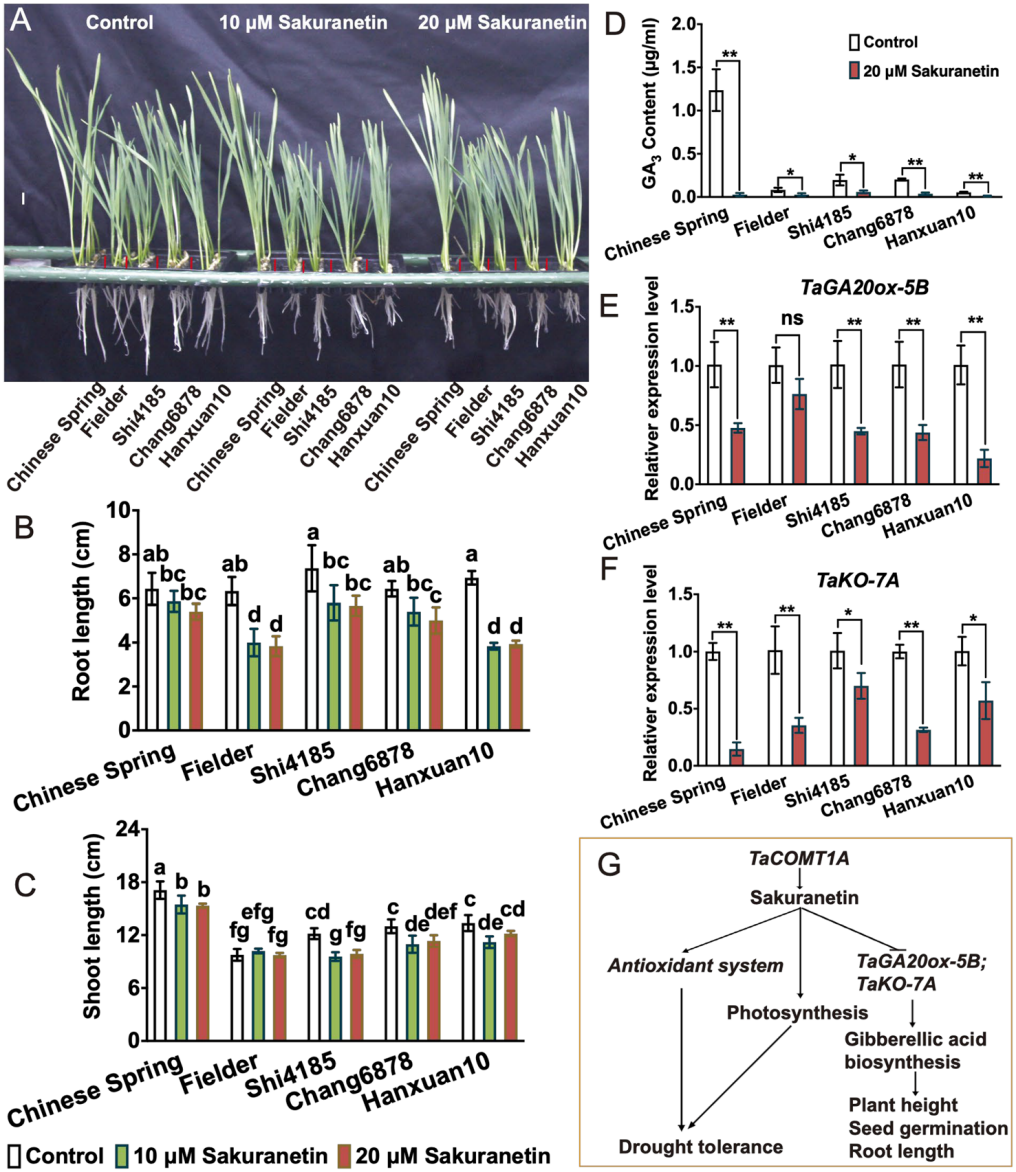
Sakuranetin application reduces shoot growth and gibberellin content while inducing drought‐responsive gene expression in wheat seedlings. (A) Representative images of five wheat cultivars grown hydroponically for 7 days in water (Control), 10 μmol sakuranetin, or 20 μmol sakuranetin. Scale bar = 1 cm. (B, C) Quantification of root length (B) and shoot length (C) from the experiment shown in (A). Data are means ± SD (*n* = 3). Different lowercase letters above bars indicate statistically significant differences among treatments for each cultivar (*p* < 0.05, one‐way ANOVA with Duncan's test). (D) Endogenous gibberellic acid (GA_3_) levels in shoots of seedlings treated with or without 20 μmol sakuranetin for 7 days, as measured by HPLC. (E–G) Relative expression levels of two gibberellin biosynthesis genes, *TaKO‐7A* (E) and *TaGA20ox‐5B* (F), in shoots following 20 μmol sakuranetin treatment. Expression was normalised to the water control for each cultivar. (G) Proposed model summarising the role of *TaCOMT1A* in wheat: Drought‐induced *TaCOMT1A* expression promotes sakuranetin biosynthesis, which enhances drought resistance and modulates plant architecture, potentially via hormonal and antioxidant pathways. Data are means ± SD (*n* = 3). Asterisks denote significant differences from the respective control within each cultivar (Student's *t*‐test: *p < 0.05, **p < 0.01; ns, not significant).

Consistent with the semi‐dwarf phenotype of *TaCOMT1A*‐OE lines, exogenous sakuranetin treatment significantly inhibited shoot and root elongation in seedlings of multiple cultivars (Figure 6A–C). Hormonal analysis confirmed that bioactive GA_3_ content was significantly reduced in both OE lines (Figure S13B) and sakuranetin‐treated plants (Figure 6D).

Transcript analysis showed that key GA biosynthetic genes (*TaGA20ox‐5B* and *TaKO‐7A*) were significantly downregulated in OE lines (Figure S13E,F) and sakuranetin‐treated plants (Figure 6E,F). Exogenous GA₃ application partially rescued the dwarfing phenotype (Figure S13A–D) and delayed seed germination of OE lines (Figure S14). *TaCOMT1A*‐OE seeds showed reduced germination rate at 12 h of imbibition, which was partially restored by 10–20 μmol GA_3_ (Figure S14A–D). Germination kinetics further confirmed that GA_3_ supplementation alleviated the germination delay in OE lines (Figure S14E–G).

In summary, sakuranetin enhances drought tolerance through three interconnected mechanisms: (1) protecting photosynthetic capacity, (2) orchestrating ABA/JA signalling to balance stress response and growth, and (3) activating drought‐related transcription factors. Concurrently, sakuranetin suppresses GA biosynthesis to restrict excessive vegetative growth, promoting a compact architecture that enhances lodging resistance (Figure 6G). The TaCOMT1A–sakuranetin module thus integrates stress adaptation and growth regulation to improve wheat yield stability under drought.

## 3 Discussion

### 3.1 TaCOMT1A Acts as a Multifunctional O‐Methyltransferase Shaping Phenylpropanoid Metabolism and Field Agronomic Traits

Phenylpropanoid metabolism serves as a central hub integrating secondary metabolism, plant architecture, and stress adaptation in crops. Our study establishes that wheat *TaCOMT1A* functions as a pivotal regulatory enzyme at a key metabolic branch point, directing carbon flux toward distinct downstream pathways in a tissue‐dependent manner—ultimately modulating both stress tolerance and field performance. Lignin and flavonoid biosynthesis share phenylalanine as the initial precursor (Zhang et al. 2021), and we observed a significant elevation in stem lignin content in *TaCOMT1A*‐overexpressing (OE) lines (Figure S3F). This indicates that in stem vascular tissues, TaCOMT1A preferentially drives metabolic flux into the monolignol pathway for S‐lignin synthesis, a conserved function widely characterised in plant COMT family members. Concurrently, TaCOMT1A facilitates the production of sakuranetin—a stress‐responsive flavonoid. Such divergent metabolic partitioning across tissues is likely governed by endogenous substrate availability and cell‐type‐specific transcriptional regulators, enabling TaCOMT1A to coordinate two agriculturally valuable field traits, including enhanced drought tolerance mediated by sakuranetin, and improved stem mechanical strength and lodging resistance resulting from lignification (Figure S3G,H).

Biochemical characterisation verified that recombinant TaCOMT1A catalyses two distinct methylation reactions: The conversion of naringenin to sakuranetin and N‐acetylserotonin (NAS) to melatonin (Figure 3). Consistent with these in vitro activities, *TaCOMT1A* overexpression markedly increased endogenous sakuranetin and melatonin contents in wheat (Figure 3C–E; Figure S6B). Functional divergence of O‐methyltransferases (OMTs) exists across cereal species: In rice, sakuranetin biosynthesis from naringenin is exclusively catalysed by naringenin 7‐O‐methyltransferase (NOMT), not canonical COMT proteins (Murata et al. 2020; Rakwal et al. 2000; Shimizu et al. 2012; Yang et al. 2021). Naringenin can undergo further hydroxylation and methylation modifications to generate diverse flavonoids, including apigenin, kaempferol, quercetin, tricin, and anthocyanins (Murata et al. 2020).

Plant OMTs are classified into two subfamilies based on molecular weight and cation dependence: Type I OMTs (23–27 kDa) require cations for activity, while Type II OMTs (38–43 kDa) function independently of cations, with both types transferring methyl groups from S‐adenosylmethionine (SAM) to hydroxyl groups of various substrates (Bureau et al. 2007; Lu et al. 2022). Phylogenetic analysis of wheat COMTs, rice COMTs, and rice NOMT revealed that TaCOMT1A clusters closely with rice COMT homologues but is distantly related to OsNOMT (Figure S7). Plant COMTs are well known for their broad substrate selectivity, catalysing the methylation of multiple phenolic compounds such as caffeic acid and 5‐hydroxyconiferaldehyde (Li et al. 2022). Integrating biochemical and phylogenetic evidence, we confirm that TaCOMT1A is a multifunctional Type II OMT in wheat, capable of producing both sakuranetin and melatonin from distinct substrates.

We also observed a genotype‐dependent response to exogenous sakuranetin among wheat cultivars with contrasting drought tolerance. The drought‐sensitive cultivar ‘Shi4185’ maintains low basal *TaCOMT1A* expression (Figure 4I), leading to the most prominent phenotypic improvement upon sakuranetin application. In contrast, drought‐tolerant cultivars ‘Chang6878’ and ‘Hanxuan10’—with inherently higher *TaCOMT1A* expression and endogenous sakuranetin levels—exhibit limited additional benefits. This variation does not reflect differences in cultivar sensitivity to sakuranetin itself, but arises from the pre‐activated state of the endogenous sakuranetin‐mediated drought tolerance pathway in drought‐adapted genotypes.

### 3.2 The TaCOMT1A–Sakuranetin Module Balances Antioxidant Defence and Gibberellin Homeostasis to Improve Drought Tolerance and Field Yield

Drought stress triggers excessive reactive oxygen species (ROS) accumulation, causing oxidative damage to cell membranes, proteins, and the photosynthetic apparatus. Our results (Figure 4G,H; Figure S9N,O) confirm that sakuranetin effectively alleviates drought‐induced oxidative stress in wheat. The TaCOMT1A–sakuranetin regulatory module activates and reinforces the enzymatic antioxidant system, with catalase (CAT) as a key component. This enhanced antioxidant capacity maintains cellular integrity, protects photosynthetic function, and reduces the accumulation of malondialdehyde (MDA)—a marker of lipid peroxidation. Rather than a passive by‐product of improved water status, antioxidant defence acts as an active, foundational component of the drought tolerance mechanism. As a natural flavonoid phytoalexin, sakuranetin also possesses intrinsic radical‐scavenging activity, further strengthening plant stress resistance.

Beyond redox regulation, the TaCOMT1A–sakuranetin module modulates gibberellin (GA) metabolism to reshape plant growth architecture. Both *TaCOMT1A* overexpression and exogenous sakuranetin treatment reduce endogenous GA_3_ content and repress the expression of key GA biosynthetic genes (*TaGA20ox‐5B* and *TaKO‐7A*), leading to reduced plant height, shortened root length, and delayed seed germination (Figure 6; Figure S13E,F). Multiple lines of evidence from field and greenhouse trials confirm that GA suppression is tightly coupled with enhanced drought tolerance (Figures 1, 2 and 4). This regulatory pattern aligns with previous studies on GA methyltransferases (GAMTs), which promote GA degradation and simultaneously improve plant drought resistance (Nir et al. 2014; Varbanova et al. 2007).

Transcriptomic analysis (Figure 5; Figure S11) further uncovered the global regulatory network underlying sakuranetin‐mediated drought tolerance. Sakuranetin stabilised photosynthetic pathways (e.g., chlorophyll biosynthesis, photosystem components) under drought, which explains the maintained Fv/fm and chlorophyll content in treated plants. Additionally, sakuranetin dynamically modulated hormone signalling: Upregulating ABA/JA pathway genes under well‐watered conditions to prime stress responses, while downregulating them under drought to avoid over‐activation and growth inhibition (Dataset S6). The coordinated upregulation of drought‐related transcription factors (WRKY, MYB, NAC, DREB; Figure S12) further amplifies the stress response, forming a multi‐layered regulatory cascade that integrates metabolic, hormonal, and transcriptional reprogramming.

We herein propose a dual regulatory model for the TaCOMT1A–sakuranetin module in wheat: First, it enhances antioxidant defence to eliminate ROS and maintain cellular homeostasis under drought; second, it modulates GA homeostasis to restrict redundant vegetative growth. This growth‐defence trade‐off strategy enables wheat to allocate more resources to stress adaptation. Most importantly, this module does not incur yield penalties under favourable growth conditions. Field trials across multiple locations and years (Shunyi, Beijing, 2022/2023; Linfen, Shanxi, 2025) demonstrated that the TaCOMT1A–sakuranetin pathway stabilises grain yield under drought stress while maintaining normal productivity under well‐watered conditions (Figures 1, 2; Figures S2, S4). Such superior performance makes this module an ideal target for breeding climate‐resilient wheat varieties adapted to fluctuating field environments. Further research is required to dissect the direct molecular link between sakuranetin and GA signalling pathways.

### 3.3 Sakuranetin as a Novel, Eco‐Friendly Biostimulant for Wheat Improvement

Against the backdrop of global climate change and frequent drought disasters, developing drought‐tolerant crop cultivars and exploring green agricultural regulators have become core strategies to ensure food security. Traditional synthetic plant growth regulators such as paclobutrazol (PBZ) are widely used to reduce plant height and mitigate drought damage in crops (Davari et al. 2021; Maheshwari et al. 2022). PBZ‐induced GA deficiency also improves lodging resistance in rice (Plaza‐Wüthrich et al. 2016). However, synthetic chemicals may pose potential risks to ecological and food safety.

As a plant‐derived flavonoid phytoalexin, sakuranetin confers resistance to a wide range of pathogens and pests (Shimizu et al. 2012). Our field and greenhouse data demonstrated that sakuranetin effectively improves wheat drought tolerance and optimises plant architecture (Figures 1, 4 and 6). Compared with synthetic PBZ, sakuranetin exhibits distinct advantages: It is low‐toxic, environmentally friendly, and confers dual resistance to both biotic and abiotic stresses (De Diego and Spíchal 2022; González‐Pérez et al. 2022). Field trials confirmed that exogenous sakuranetin application increases wheat yield by 13.22%–18.6% under drought (Figure 1), highlighting its potential as a novel eco‐friendly biostimulant for large‐scale agricultural use.

From a molecular breeding perspective, *TaCOMT1A* offers multiple practical solutions for wheat improvement. First, natural allelic variations and expression quantitative trait loci (eQTLs) of *TaCOMT1A* can be developed into functional molecular markers for marker‐assisted selection, enabling the pyramiding of drought tolerance, lodging resistance, and high‐yield traits. Second, targeted overexpression of *TaCOMT1A* in commercial elite wheat varieties—validated by our transgenic field data (Figure 2; Figure S4)—provides a feasible transgenic approach to create new drought‐tolerant lines with stable field performance. Third, manipulation of the endogenous sakuranetin metabolic pathway offers a new direction for genome editing‐based crop improvement.

Notably, the TaCOMT1A–sakuranetin module's ability to maintain yield under both well‐watered and drought conditions addresses a critical limitation of many stress‐tolerance traits, which often incur yield costs in favourable environments. By coordinating growth (via GA suppression) and defence (via antioxidant and transcriptional activation), this module enables wheat to allocate resources efficiently, ensuring productivity across variable environments. Future research should focus on dissecting the direct molecular link between sakuranetin and GA signalling, as well as evaluating the efficacy of sakuranetin in combination with other biostimulants for enhanced stress protection.

In conclusion, our study delineates a complete regulatory cascade—from *TaCOMT1A* (multifunctional enzyme) to sakuranetin/melatonin (key metabolites) to coordinated physiological and transcriptional responses—that improves wheat drought tolerance, lodging resistance, and yield stability. This work not only advances our mechanistic understanding of phenylpropanoid metabolism in crop stress adaptation but also provides elite gene resources and eco‐friendly biostimulant candidates for developing high‐yield, drought‐resilient wheat—with broad application prospects in modern sustainable agriculture.

## 4 Materials and Methods

### 4.1 Plant Materials

The following hexaploid wheat ( *Triticum aestivum* ) genotypes were used in this study: The conventional cultivars ‘Chinese Spring’, ‘Shi4185’, ‘Chang6878’, and ‘Hanxuan10’; the transgenic receptor cultivar ‘Fielder’; those cultivars were obtained from the Institute of Crop Sciences in the Chinese Academy of Agricultural Sciences; and two allelic ethyl methanesulfonate (EMS)‐induced mutant lines (E829 and E830) of the *TaCOMT1A* gene (*Traes_1AL_D9035D5E0.1*) in the ‘Jing411’ background (mutant library stock IDs: 455829 and 455 830, obtained from http://jing411.molbreeding.com).

### 4.2 Growth Conditions

Unless otherwise stated, seedlings were cultivated in a controlled‐environment growth chamber under the following conditions: Photoperiod (16‐h light/8‐h dark), light intensity (350 μmol m^−2^ s^−1^), temperature (22°C day/18°C night), and relative humidity (60%–70%).

### 4.3 Vector Construction and Wheat Transformation

The coding sequence of *TaCOMT1A* (*Traes_1AL_D9035D5E0*) was cloned into the binary vector pWMB190 under the control of the maize ubiquitin promoter (Figure S1A). The recombinant plasmid was introduced into *Agrobacterium tumefaciens* strain GV3101 and transformed into the wheat cultivar ‘Fielder’ using an established protocol (Zhou et al. 2022). Primer sequences are listed in Table S1.

### 4.4 Drought Stress Treatments at the Seedling Stage

For soil‐based drought assays, a 1:2 (g/mL) mixture of peat‐based substrate (Pindstrup) and water was prepared. Germinated seeds of ‘Fielder’ and transgenic lines were sown in shallow basins containing 1.8 kg of this mixture. Seedlings were watered on day 1 after sowing and received a second irrigation (1 L per basin) on day 14. For drought stress, watering was withheld from day 1. Shoot tissue from six seedlings constituted one biological replicate and was flash‐frozen in liquid nitrogen for subsequent analysis.

### 4.5 Chemical Treatment and Drought Stress Assay

For chemical treatment, sakuranetin was dissolved and thoroughly mixed into the soil medium at final concentrations of 10 or 20 μmol prior to sowing. Drought stress was initiated from day 1 post‐sowing by withholding water. A single recovery irrigation (500 mL per basin) was applied at the end of the stress period on day 15 (for experiments in Figure 1 and Figure S6), day 21 (for experiments in Figure S7), or day 28 (for experiments in Figure 4). Seedlings were harvested for phenotypic observation and physiological analysis at the following time points: Day 12 (corresponding to Figure 1 and Figure S6), day 21 (corresponding to Figure S7), or day 28 (corresponding to Figure 4) after sowing.

For hydroponic culture, seedlings of multiple cultivars were grown in water (control), 10 μmol, or 20 μmol sakuranetin or GA_3_ solution for seven days. Shoot and root lengths were measured, and tissue was collected for hormone and RNA analysis.

### 4.6 Pot Specifications

Experiments were conducted using three types of plastic basins, including white basins (for long‐term assays): 60 cm (L) × 41 cm (W) × 6.5 cm (H); blue basins (for medium‐term assays): 18 cm (L) × 10 cm (W) × 5.5 cm (H); and green basins (for specific mutant assays): 33 cm (L) × 26 cm (W) × 3.5 cm (H).

### 4.7 Field Trial Design and Management

Field experiments were conducted in a suburb of Beijing (40°13′49″ N, 116°33′28″ E) and Linfen, Shanxi (39°75′19″ N, 114°51′98″ E). The trials employed a randomised block design with three replications. Each plot measured 1.5 m × 8 m (12 m^2^) with six rows and a sowing density of 120 kg/ha. Two irrigation regimes were imposed: Limited Irrigation (LIR; pre‐wintering and jointing stages) and Well Irrigated (WIR; pre‐wintering, jointing, and filling stages). Each irrigation event applied 750 m^3^ of water per hectare.

### 4.8 Application of Sakuranetin

A 10 mM stock solution of sakuranetin (Cat# SML0573, Sigma‐Aldrich) was prepared in ethanol and stored at −20°C. Working solutions for soil (10, 20 μmol) or foliar application (100 μmol) for field treatments were prepared by diluting the stock in water containing 0.02% (v/v) Tween‐20, with final ethanol concentrations not exceeding 1%. The solution was stirred vigorously for 10 min before application to ensure homogeneity. An equivalent solution of 1% ethanol and 0.02% Tween‐20 served as the mock control. Solutions were applied within 4 h of preparation using a backpack sprayer until run‐off; 100 μmol sakuranetin solution was sprayed at a volume of 450 L/ha at the jointing stage with two foliar applications.

### 4.9 Physiological and Biochemical Assays

The activities of catalase (CAT) and the contents of hydrogen peroxide (H_2_O_2_) and malondialdehyde (MDA) were quantified using commercial assay kits (Comin Biotechnology). Chlorophyll was extracted with 80% acetone and measured spectrophotometrically. Lignin content was determined using a plant lignin ELISA kit (Jianglai). Absorbance was read on a Varioskan LUX microplate reader (Thermo Scientific).

### 4.10 Agronomic Trait and Stem Strength Evaluation

For field‐grown plants, 15 individual plants per line were randomly selected for trait measurement. Data collected included grain weight per plant, thousand‐seed weight, seed length, seed width, and plant height. The stress susceptibility index (SSI), stress tolerance index (STI), and geometric mean productivity (GMP) were calculated as described (Zhou et al. 2020). Stem strength (puncture resistance and lodging resistance) was measured at maturity using a handheld digital force gauge (Handpi, HP‐200) with specialised probes. Chlorophyll content was estimated as SPAD values using a SPAD‐502Plus chlorophyll meter. The maximum quantum yield of photosystem II (F_v_/F_m_) was determined on dark‐adapted leaves using a portable fluorometer (FluorPen FP 100).

### 4.11 Recombinant Protein Production and Enzyme Assays

The *TaCOMT1A* coding sequence was cloned into pET‐28a (EcoRI/SalI) for expression in *E. coli* . The His‐tagged recombinant protein was purified via nickel‐affinity chromatography. Protein concentration was determined by the Bradford method (Bio‐Rad). Enzyme kinetics were analysed by incubating 50 μg of purified TaCOMT1A with varying concentrations (0.1–1.6 mM) of N‐acetylserotonin (NAS) or naringenin in reaction buffer at 37°C for 60 min. The products (melatonin or sakuranetin) were quantified by HPLC. Kinetic parameters (K_m_, V_max_) were derived from Lineweaver‐Burk plots.

### 4.12 Metabolite Profiling and Quantification

For targeted analysis, sakuranetin and melatonin in enzyme reactions were quantified via HPLC (Wooking K2025) with detection at 270 nm and 220 nm, respectively (Figure S8). For broad profiling, metabolites from ‘Fielder’ and transgenic line #9 field samples were extracted and analysed by UPLC‐MS/MS (SHIMADZU CBM30A system coupled to an Applied Biosystems 4500 QTRAP) at Metware Biotechnology Inc. (Wuhan, China). A total of 158 flavonoids were identified.

### 4.13 RNA Sequencing and Transcriptome Analysis

Total RNA was extracted from 36 samples comprising three replicates of four treatments across three cultivars (‘Shi4185’, ‘Chang6878’, ‘Hanxuan10’). Treatments were: Well‐watered (W), Well‐watered +20 μmol sakuranetin (SW), Drought (D), and Drought +20 μmol sakuranetin (SD). Library construction, sequencing, and bioinformatic analysis were performed by Oebiotech Co. Ltd. (Shanghai). Differential expression genes (DEGs) were identified using EBseq (FDR < 0.05, |fold‐change| ≥ 1.5). Functional annotation was performed using GO, KEGG, Pfam, Swiss‐Prot, eggNOG, NR, and KOG databases.

### 4.14 RNA Extraction and Quantitative qRT‐PCR

Total RNA was extracted with a Plant Total RNA Kit (Zoman Biotechnology) and reverse‐transcribed using a Fast Quant RT Kit (Vazyme). Quantitative PCR was performed using SuperReal PreMix (Vazyme) on a real‐time PCR system. The wheat *TaActin* gene (*TraesCS1A01G274400*) served as the internal control. The data analysis method is the 2^^(ΔΔCt)^ method. Primer sequences are in Table S1.

### 4.15 Phylogenetic Analysis

Amino acid sequences of 12 wheat COMT proteins (Phytozome) and 2 rice COMT proteins (National Rice Data Center) were aligned. A phylogenetic tree was constructed using the neighbour‐joining method in MEGA11 software with 1 000 bootstrap replicates.

### 4.16 Statistical Analysis

Data are presented as the mean ± standard deviation (SD). Statistical significance between two groups was assessed using a two‐tailed Student's *t*‐test. For comparisons among multiple groups, a one‐way analysis of variance (ANOVA) followed by Duncan's multiple range test was performed using the Statistical Package for the Social Sciences (SPSS) 17.0 statistical software package. Differences were considered significant at *p < 0.05, **p < 0.01. All analyses were based on at least three independent biological replicates. Graphs were generated using GraphPad Prism (Version 8.4.0), and figures were assembled with Adobe Illustrator.

## Author Contributions

Conceptualisation: M.L., M.C., W.T., C.X., W.Y., and Y.Z. Methodology: M.L., K.C., J.G., Q.W., J.C., Z.X., P.D., C.X. and W.Y. Validation: M.L., K.C., J.G., Q.W., J.C., Z.X., P.D., C.X. and W.Y. Data curation: M.L., K.C., J.G., Q.W., J.C., Z.X., P.D., C.X. and W.Y. Investigation: M.L., W.T., J.G., Q.W., C.X., and W.Y. Formal analysis: M.L., K.C., J.G., Q.W., J.C., Z.X., P.D., C.X. and W.Y. Resources: M.L., W.T., K.C., J.G., Q.W., J.C., Z.X., P.D., C.X. and W.Y. Project administration: M.L., C.X. and W.Y. Visualisation: M.L., K.C., J.G., Q.W., J.C., Z.X., P.D., Y.M., C.X. and W.Y. Supervision and Funding acquisition: M.L., M.C., W.T., and Y.M. Writing (original draft): M.L., M.C., C.X. and W.Y. Writing (review and editing): M.L., M.C., W.T., P.D., Y.M., C.X. and W.Y.

## Funding

National Key Research and Development Program of China (2022YFD1200202). Innovation Program of Chinese Academy of Agricultural Sciences (CAAS‐CSCB‐202401).

## Conflicts of Interest

The authors declare no conflicts of interest.

## Supporting information


**Figure S1:** Generation and molecular identification of *TaCOMT1A*‐overexpressing transgenic wheat.
**Figure S2:** *TaCOMT1A* Agronomic Traits of Wild‐Type and TaCOMT1A‐Overexpressing Wheat Lines under Well‐Irrigated and Limited Irrigation Conditions in 2023.
**Figure S3:** *TaCOMT1A* TaCOMT1A overexpression improves lodging resistance and modifies stem architecture at maturation.
**Figure S4:** *TaCOMT1A* TaCOMT1A overexpression enhances drought tolerance at the seedling stage.
**Figure S5:** *TaCOMT1A* ROS staining in ‘Fielder’ and TaCOMT1A overexpression lines under drought conditions.
**Figure S6:** Analysis of TaCOMT1A activity in melatonin biosynthesis.
**Figure S7:** Phylogenetic analysis of the COMT protein family in wheat and rice.
**Figure S8:** UPLC‐MS/MS analysis of sakuranetin and melatonin in standard samples and enzymatic reactions.
**Figure S9:** Enhancement of drought tolerance by exogenous sakuranetin.
**Figure S10:** Loss of TaCOMT1A function reduces drought tolerance, which is rescued by exogenous sakuranetin application.
**Figure S11:** Transcriptome analysis of wheat cultivars in response to drought and exogenous sakuranetin.
**Figure S12:** Sakuranetin modulates the expression of drought‐responsive transcription factors in wheat.
**Figure S13:** *TaCOMT1A* GA3 complementation and biosynthetic gene expression in TaCOMT1A‐overexpressing wheat.
**Figure S14:** *TaCOMT1A* TaCOMT1A overexpression delays seed germination, partially rescued by exogenous GA3.
**Table S1:** Primers used for PCR in this study.
**Table S2:** Effects of Exogenous Sakuranetin Application on Yield and Agronomic Traits in Wheat (Triticum aestivum cv. Shi4185) under Well‐Irrigated and Limited Irrigation Conditions (Shunyi, Beijing, 2024).
**Table S3:** Effects of Exogenous Sakuranetin Application on Yield and Agronomic Traits in Wheat (Triticum aestivum cv. Shi4185) under Well‐Irrigated and Limited Irrigation Conditions (Linfen, Shanxi, 2025).
**Table S4:** *TaCOMT1A* Agronomic Performance of Wild‐Type and TaCOMT1A‐Overexpressing Wheat Under Well‐Irrigated Field Conditions (Shunyi, Beijing, 2022).
**Table S5:** *TaCOMT1A* Agronomic Performance of Wild‐Type and TaCOMT1A‐Overexpressing Wheat Under Limited‐Irrigation Field Conditions (Shunyi, Beijing, 2022).
**Table S6:** *TaCOMT1A* Agronomic Performance of Wild‐Type and TaCOMT1A‐Overexpressing Wheat Under Well‐Irrigated Field Conditions (Shunyi, Beijing, 2023).
**Table S7:** *TaCOMT1A* Agronomic Performance of Wild‐Type and TaCOMT1A‐Overexpressing Wheat Under Limited‐Irrigation Field Conditions (Shunyi, Beijing, 2023).

**Data S1:** 158 Flavonoid content in ‘Fielder’ and overexpression line *Ubi:TaCOMT1A #9*.
**Data S2:** Summary of transcriptomic data in three wheat cultivars, i.e., ‘Shi4185’, ‘Chang6878’, and ‘Hanxuan10’.
**Data S3:** Differential expression genes (DEGs) analysis in wheat cultivar ‘Shi4185’.
**Data S4:** Differential expression genes (DEGs) analysis in wheat cultivar ‘Chang6878’.
**Data S5:** Differential expression genes (DEGs) analysis in wheat cultivar ‘Hanxuan10’.
**Data S6:** Differential expression genes (DEGs) related to sakuranetin regulation of drought tolerance in three wheat cultivars, i.e., ‘Shi4185’, ‘Chang6878’, and ‘Hanxuan10’.

## Data Availability

The data that supports the findings of this study are available in the Supporting Information of this article.
