# Supplementary material for: The TaCOMT1A‐Synthesised Sakuranetin Is a Novel Biostimulant Enhancing Drought Tolerance and Yield in Wheat
Source: Plant Biotechnol J. 2026 Aug 7:10.1111/pbi.70734. Online ahead of print. doi: 10.1111/pbi.70734 (PMC13449750; doi:10.1111/pbi.70734)
Supplement: Supplementary file 1 [file PBI-9999-0-s002.docx]

**The TaCOMT1A-synthesized sakuranetin enhances drought** **tolerance and reduces plant height in wheat**

Mingzhao Luo^1, 2, 3 *^, Chengjie Xu^1, 4, *^, Wenjing Yang^5, *^, Wensi Tang^1^, Kai Chen^1^, Jiaqing Guo^1^, Qiyu Wang^1, 4^, Jun Chen^1^, Zhaoshi Xu^1^, Pierre Delaplace^3^, Youzhi Ma^1^, Yongbin Zhou^1, †^, Ming Chen^1, †^

^1^ State Key Laboratory of Crop Gene Resources and Breeding, Key Laboratory of Biology and Genetic Improvement of Triticeae Crops, Ministry of Agriculture, Institute of Crop Sciences, Chinese Academy of Agricultural Sciences (CAAS), 100081, Beijing, China.

^2^ Tianjin Crop Research Institute, Tianjin Academy of Agricultural Sciences, 300384, Tianjin, China.

^3^ Plant Genetics and Rhizosphere Processes laboratory, Gembloux Agro-Bio Tech, Teaching and Research Centre (TERRA), University of Liège, B-5030, Gembloux, Belgium.

^4^ Key Laboratory of Sustainable Dryland Agriculture, College of Agriculture, Shanxi Agricultural University, 030801, Jinzhong, China.

^5^ Key Laboratory of Grain Crop Genetic Resources Evaluation and Utilization, Institute of Crop Sciences, Chinese Academy of Agricultural Sciences, Beijing 100081, China.

Email address:

Chengjie Xu: xu1253824860@163.com; Wenjing Yang: ywj103628@163.com; Wensi Tang: tang_wensi@yeah.net; Kai Chen: chenkai8@vip.163.com; Jiaqing Guo: 15843481863@139.com; Qiyu Wang: wangqiyu0568@163.com; Jun Chen: chenjun01@caas.cn; Zhaoshi Xu: xuzhaoshi@caas.cn; Pierre Delaplace: pierre.delaplace@uliege.be; Youzhi Ma: mayouzhi@caas.cn.

^*^ These authors contributed equally to this work.

^†^ Correspondence author:

chenming02@caas.cn and zhouyongbin@caas.cn

**Figure S1. Generation and molecular identification of *TaCOMT1A*-overexpressing transgenic wheat.**

(A) Schematic of the plant transformation vector pWMB190-*Ubi:TaCOMT1A*. The *TaCOMT1A* coding sequence is under the control of the maize ubiquitin (Ubi) promoter. (B) PCR confirmation of *TaCOMT1A* transgene integration in genomic DNA from T_3_ generation plants. A 1069-bp fragment specific to the transgene was amplified. M, DNA molecular weight marker; lanes 2–9, independent *Ubi:TaCOMT1A* transgenic lines; lane 10, F, wild-type ‘Fielder’; lane 11, N, no-template control. (C) Relative expression of *TaCOMT1A* in the leaves of T_3_ transgenic lines and wild-type ‘Fielder’ under normal conditions. Expression was analyzed by qRT-PCR and normalized to the TaActin reference gene. Data are mean ± SD (n = 3 biological replicates). Asterisks indicate a significant difference from the wild type (Student’s t-test, **p< 0.01).

**Figure S2. Agronomic Traits of Wild-Type and *TaCOMT1A*-Overexpressing Wheat Lines under Well-Irrigated and Limited Irrigation Conditions in 2023.**

(A–J) Measured traits of wild-type ‘Fielder’ and *TaCOMT1A* overexpression lines under well-irrigated (WIR) and limited irrigation (LIR) conditions: seed length (A), seed width (B), thousand seed weight (C), biomass (D), plant height (E), grain weight per plant (F). Consistent with the 2022 season, plants were sown at 50 seeds per 2-meter row. Each genotype was allocated three rows per replicate, with three independent replicates per irrigation treatment. LIR: Two irrigations (pre-wintering and jointing stages). WIR: Three irrigations (pre-wintering, jointing, and filling stages). Each irrigation supplied 750 m^3^ of water per hectare. Data are presented as mean ± SD (n = 15 plants per genotype per treatment). Asterisks indicate significant differences between a transgenic line and ‘Fielder’ under the same irrigation condition (Student’s t-test: *P < 0.05, **P < 0.01).

**Figure** **S3. *TaCOMT1A* overexpression improves lodging resistance and modifies stem architecture at maturation.**

(A) Representative image illustrating enhanced lodging resistance in transgenic lines. (B) Whole-plant phenotype of ‘Fielder’ and *TaCOMT1A* overexpression lines. (C) Morphology of the main spike and stem internodes. (D) Length of individual stem internodes. (E) Diameter at the widest point of each internode. (F) Stem lignin content. (G, H) Quantitative assessment of stem mechanical strength: puncture resistance (G) and lodging resistance capacity (H).

Data are presented as mean ± SD (n = 3 biological replicates). Asterisks indicate significant differences between transgenic lines and the ‘Fielder’ wild type (Student’s t-test: *P < 0.05, **P < 0.01). Scale bars (in A and C): 5 cm.

**Figure S4.** ***TaCOMT1A* overexpression enhances drought tolerance at the seedling stage.**

(A) Representative phenotype of 7-day-old wild-type ‘Fielder’ and *TaCOMT1A*-overexpressing (OE) lines. Seedlings were grown in a soil: water mixture (1:2, w/v); watering was withheld from Day 1 after drought (DAD). (B) Phenotype of seedlings after 10 days of drought stress. (C) Phenotype following 15 days of drought stress and 7 days of rewatering. (D) Survival rate quantified after the drought-rewatering cycle shown in (C). (F–H) Physiological parameters after 12 days of drought: (E) shoot fresh weight and (F) shoot dry weight. (I, J) Stress markers after 12 days of drought: (G) malondialdehyde (MDA) and (H) hydrogen peroxide (H_2_O_2_) content. (I) Catalase (CAT) enzyme activity after 12 days of drought. Bar graphs show mean ± SD (n = 3 biological replicates, with 6 seedlings per replicate). Asterisks in (D–I) denote significant differences between each OE line and the wild-type at the same time point (Student’s t-test, *p < 0.05, **p < 0.01). Scale bars = 3 cm.

**Figure S5. ROS staining in ‘Fielder’ and *TaCOMT1A* overexpression lines under drought conditions.**

(A) DAB staining for H_2_O_2_ and (B) NBT staining for O_2_^-^ in leaves of wild-type ‘Fielder’ and three independent *TaCOMT1A*-OE lines following drought treatment. The reduced intensity of stain in the OE lines demonstrates that *TaCOMT1A* overexpression mitigates stress-induced ROS accumulation.

**Figure S6. Analysis of TaCOMT1A activity in melatonin biosynthesis.**

(A) *In vitro* enzyme kinetics of TaCOMT1A with N-acetylserotonin (NAS). Michaelis-Menten plot showing the activity of purified TaCOMT1A (1 µg) incubated for 60 min at 37°C with varying concentrations of NAS and 200 µmol S-adenosylmethionine (SAM). Melatonin production was quantified by UPLC-MS/MS. Kinetic parameters (K_m_ and V_max_) were derived from nonlinear regression. (B) Endogenous melatonin levels in wild-type and transgenic plants. Melatonin content was quantified by UPLC-MS/MS in the shoots of wild-type ‘Fielder’ and three independent *TaCOMT1A*-overexpressing lines (#6, #8, #9) under control conditions. Data are presented as mean ± SD (n = 3 biologically independent samples). Significant differences from the wild-type were determined by Student’s t-test (**p< 0.01).

**Figure S7. Phylogenetic analysis of the COMT protein family in wheat and rice.**

The evolutionary tree was constructed using the full-length amino acid sequences of 12 wheat COMT proteins (retrieved from Phytozome, [https://phytozome-next.jgi.doe.gov](https://phytozome-next.jgi.doe.gov/)) and 2 rice COMT proteins (retrieved from the National Rice Data Center, <https://www.ricedata.cn/gene/>). The tree was inferred using the neighbor-joining or maximum likelihood method. Red star: TaCOMT1A protein characterized in this study.

**F****igure S8. UPLC-MS/MS analysis of sakuranetin and melatonin in standard samples and enzymatic reactions**

(A, B) UPLC-MS/MS chromatograms of authentic standards for sakuranetin (A) and melatonin (B), confirming detection and quantification parameters. (C, D) Representative time-course chromatograms from a single kinetic reaction, showing the formation and accumulation of sakuranetin (C) and melatonin (D) peaks.

**Figure S9. Enhancement of drought tolerance by exogenous sakuranetin.**

(A–C) Phenotype of five wheat cultivars after 7 days of drought stress. Seeds were sown in a soil: liquid mixture (1 g soil: 2 mL solution) containing 0 µmol (A, water control), 10 µmol (B), or 20 µmol (C) sakuranetin. Water was withheld from day 1 after drought (DAD). Cultivars are grouped as drought-sensitive (DS: ‘Chinese Spring’, ‘Fielder’, ‘Shi4185’) or drought-tolerant (DT: ‘Chang6878’, ‘Hanxuan10’). (D–F) Phenotype after 10 days of drought under 0 µmol (D), 10 µmol (E), or 20 µmol (F) sakuranetin. (G–I) Survival after 15 days of drought followed by 7 days of rewatering under 0 µmol (G), 10 µmol (H), or 20 µmol (I) sakuranetin. (J) Soil relative water content (RWC) was monitored during the drought period. (K) Quantified survival rates after the drought–recovery cycle are shown in (G–I). (L, M) Shoot relative water content (L) and fresh weight (M) after 10 days of drought. (N, O) Oxidative stress markers after 10 days of drought: malondialdehyde (MDA, N) and hydrogen peroxide (H_2_O_2_, O) content. Data are mean ± SD (n = 3 biological replicates, with 6 seedlings per replicate). Different lowercase letters indicate significant differences among sakuranetin concentrations within a cultivar and time point (one-way ANOVA, Duncan’s test, *p < 0.05). Scale bars = 2 cm.

**Figure S10. Loss of *TaCOMT1A* function reduces drought tolerance, which is rescued by exogenous sakuranetin application.**

(A–C) Five-day-old seedlings of the wild-type (WT) cv. ‘Jing411’ and the *TaCOMT1A* EMS-induced mutant lines (E829 and E830). (D) Twenty-one-day-old seedlings of the WT and mutant lines. (E) Phenotype of WT and mutant plants sprayed with water (Mock) before a three-week drought stress period. (F) Phenotype of WT and mutant plants sprayed with 20 μM sakuranetin prior to a three-week drought stress period. (G–H) Fresh weight (G) and dry weight (H) of WT and mutant plants following the 21-day drought period. (I) Survival rates of WT and mutant plants after rehydration. The two allelic EMS mutant lines (E829 and E830) of *TaCOMT1A* (*Traes_1AL_D9035D5E0.1*) were derived from the wheat cultivar ‘Jing411’ (mutant library stock IDs: 455829 and 455830). All experiments were performed with these mutants alongside the WT control. Scale bar = 1 cm.

**Figure S11. Transcriptome analysis of wheat cultivars in response to drought and exogenous sakuranetin.**

(A) Number of differential expression genes (DEGs) (up- and downregulated) identified in four pairwise comparisons for three wheat cultivars: drought-tolerant 'Shi4185', moderate 'Chang6878', and drought-sensitive 'Hanxuan10'. (B–D) Venn diagrams showing overlaps of DEG sets from the four comparisons within each cultivar: (B) 'Shi4185', (C) 'Chang6878', (D) 'Hanxuan10'. Numbers in unique sections represent cultivar-specific DEGs; overlapping regions represent co-regulated DEGs across treatments. Key candidate gene groups are highlighted: red borders indicate DEGs potentially involved in sakuranetin-mediated drought tolerance; yellow borders indicate DEGs related to sakuranetin response under well-watered conditions. W, well-watered control; SW, well-watered + sakuranetin; D, drought; SD, drought + sakuranetin. Cultivar prefixes: S_, 'Shi4185'; C_, 'Chang6878'; H_, 'Hanxuan10'.

**Figure S12. Sakuranetin modulates the expression of drought-responsive transcription factors in wheat.**

(A–C) Heatmap showing the expression levels of key transcription factors (TFs) identified from differential expression analysis in response to sakuranetin and drought treatments across three wheat cultivars: ‘Shi4185’ (A), ‘Chang6878’ (B), and ‘Hanxuan10’ (C). Expression values are presented as log_2_ fold change relative to the well-watered control (W). Color scale indicates up-regulation (red) or down-regulation (blue). W (well-watered control), SW (well-watered + sakuranetin), D (drought), SD (drought + sakuranetin). Abbreviations: TFs, transcription factors; DEGs, differential expression genes; FC, fold change.

**Figure S13. GA_3_ complementation and biosynthetic gene expression in *TaCOMT1A*-overexpressing wheat.**

(A) Phenotype of wild-type ‘Fielder’ and *TaCOMT1A*-overexpressing lines after 7-day treatment with water (control) or 10 µmol GA_3_. (B–D) Quantification of (B) endogenous GA_3_ content, (C) shoot length, and (D) root length for plants described in (A). (E, F) Relative expression of GA biosynthetic genes (E) *TaGA20ox-5B* and (F) *TaKO-7A* in the shoots of wild-type and *TaCOMT1A*-OE lines under control conditions, as determined by qRT-PCR. Scale bar = 1 cm (panel a). Data are presented as mean ± SD (n = 3 biologically independent samples). Significant differences from the wild-type ‘Fielder’ within the same treatment group were determined by Student’s t-test (*p< 0.05, **p< 0.01).

**Figure S14. *TaCOMT1A* overexpression delays seed germination, partially rescued by exogenous GA_3_.**

(A–C) Representative images of germinating seeds from wild-type ‘Fielder’ and *TaCOMT1A*-overexpressing (OE) lines after 12-hour imbibition in (A) water (control), (B) 10 µmol GA_3_, or (C) 20 µmol GA_3_. (D) Germination rate quantified at the 12-hour time point for the treatments shown in (a-c). (E–G) Germination kinetics over time for seeds imbibed in (E) water, (F) 10 µmol GA_3_, or (G) 20 µmol GA_3_. Data are presented as mean ± SD (n = 3 biological replicates, with 50 seeds per replicate per genotype). Significant differences from the wild-type ‘Fielder’ at the same time point and treatment were determined by Student’s t-test (*p < 0.05).

Table S1. Primers for PCR were used in this study.

| **Primer name** | **Primer sequence (5′-3′)** | **Gene ID** | **Using Primer Experiments** |
| --- | --- | --- | --- |
| TaCOMT1AL-A1F | AGATGAATGGGTGGATTCGTGTG | *Traes_1AL_D9035D5E0* | Primer for gene cloning |
| TaCOMT1AL-A1R | CTCCAGAAAATGATCCAAGTAAAAT |  |  |
| TaCOMT1AL-A2F | GTGTGTGTTACAAATGGTTTAGTGA | *Traes_1AL_D9035D5E0* | Primer for gene cloning |
| TaCOMT1AL-A2R | CTTAGGAACACCGAGAATACGATGC |  |  |
| TaCOMT1AL-PF | CAAGGTGGTAGTCGTGGAG | *Traes_1AL_D9035D5E0* | Primers for positive detection |
| TaCOMT1AL-PR | AACAAATTGACGCTTAGACA |  |  |
| TaCOMT1AL-qF | ATGGAGCATGTTCCCGCAATT | *Traes_1AL_D9035D5E0* | Primer for qPCR |
| TaCOMT1AL-qR | CTATTTTGTAAATTCAATAGCCCACGATCC |  |  |
| TaGA20ox-5B-F | TTAGCTGCTGAGACTGAGAAAA | *TraesCS5B02G560300* | Primer for qPCR |
| TaGA20ox-5B-R | CCATGCTTCTTCGTACGTAGTA |  |  |
| TaKO-7A-F | GTGCAACATGAACAAGAAGGAT | *TraesCS7A02G362300* | Primer for qPCR |
| TaKO-7A-R | GAATGCCATGGTCTTGTACATG |  |  |
| TaActin1-F | AAATCTGGCATCACACTTTCTAC | *TraesCS1A01G274400* | Primer for qPCR |
| TaActin1-R | GTCTCAAACATAATCTGGGTCATC |  |  |

Table S2. Effects of Exogenous Sakuranetin Application on Yield and Agronomic Traits in Wheat (*Triticum aestivum* cv. Shi4185) under Well-Irrigated and Limited Irrigation Conditions (Shunyi, Beijing, 2024).

| **Field conditions** | **Well-irrigated (WIR)** | | | **Limited irrigation (LIR)** | | |
| --- | --- | --- | --- | --- | --- | --- |
| ‘Shi4185’ | Control | Sakuranetin | Yield increase (%) | Control | Sakuranetin | Yield increase (%) |
| Yield (kg/ha.) | 6444.44±181.40 | 7296.30±188.86 | 13.22%* | 5296.30±343.47 | 6148.15±188.86 | 16.08%* |
| Spike number per ha. (no.) | 4237647±1062957 | 4656471±609889 | 9.88% | 3796667±524330 | 4355000±525650 | 14.71%** |
| Seed number per spike (no.) | 31.68±5.20 | 34.02±4.08 | 7.39% | 32.87±1.78 | 34.90±2.40 | 6.18% |
| Thousand seed weight (g) | 44.16±1.99 | 44.34±0.47 | 0.41% | 41.00±1.20 | 41.82±1.39 | 2.00% |

The field trial was conducted using a sowing density of 120 kg/ha. Individual plots measured 1.5 m × 8 m, and each treatment (sakuranetin application vs. control) was replicated three times under both limited irrigation (LIR) and well-irrigated (WIR) conditions. Irrigation Protocol: LIR: Two irrigations were applied at the pre-wintering and jointing stages. WIR: Three irrigations were applied at the pre-wintering, jointing, and grain-filling stages. Each irrigation event applied 750 m^3^ of water per hectare. Data are presented as mean ± standard deviation (SD) (n = 15, three biological replicates). Significant differences between 100 µmol sakuranetin-treated plants and the water-only control at the same sampling point were determined using Student's t-test (**P* < 0.05; ***P* < 0.01).

Table S3. Effects of Exogenous Sakuranetin Application on Yield and Agronomic Traits in Wheat (*Triticum aestivum* cv. Shi4185) under Well-Irrigated and Limited Irrigation Conditions (Linfen, Shanxi, 2025).

| **Field conditions** | **Well-irrigated (WIR)** | | | **Limited irrigation (LIR)** | | |
| --- | --- | --- | --- | --- | --- | --- |
| ‘Shi4185’ | Control | Sakuranetin | Yield increase (%) | Control | Sakuranetin | Yield increase (%) |
| Yield (kg/ha.) | 5514.81 ± 64.52 | 5781.48 ± 165.45 | 4.61% | 1012.96 ± 23.28 | 1244.44 ± 96.55 | 18.6%* |
| Spike number per ha. (no.) | 6139640 ± 319769 | 6371371 ± 584952 | 3.64% | 3602102 ± 128118 | 4564565 ± 431634 | 21.09% |
| Seed number per spike (no.) | 43.02 ± 2.83 | 43.73 ± 0.65 | 1.64% | 27 ± 3.48 | 26.72 ± 4.13 | -1.06% |
| Thousand seed weight (g) | 41.43 ± 1.97 | 40.97 ± 4.06 | -1.12% | 29.65 ± 2.33 | 31.68 ± 4.39 | 6.41% |

The field trial was conducted using a sowing density of 120 kg/ha. Individual plots measured 1.5 m × 8 m, and each treatment (sakuranetin application vs. control) was replicated three times under both limited irrigation (LIR) and well-irrigated (WIR) conditions. Sakuranetin application for 450 L/ha (concentration is 100 µmol). LIR: Two irrigations were applied at the pre-wintering and jointing stages. WIR: Three irrigations were applied at the pre-wintering, jointing, and grain-filling stages. Each irrigation event applied 750 m^3^ of water per hectare. Data are presented as mean ± standard deviation (SD) (n = 15, three biological replicates). Significant differences between 100 µmol sakuranetin-treated plants and the water-only control at the same sampling point were determined using Student's t-test (**P* < 0.05; ***P* < 0.01).

Table S4. Agronomic Performance of Wild-Type and *TaCOMT1A*-Overexpressing Wheat Under Well-Irrigated Field Conditions (Shunyi, Beijing, 2022).

| **Traits** | **Fielder** | ***Ubi:TaCOMT1A#6*** | | ***Ubi:TaCOMT1A#8*** | | ***Ubi:TaCOMT1A#9*** | |
| --- | --- | --- | --- | --- | --- | --- | --- |
|  | Mean ± SD | Mean ± SD | Yield increase (%) | Mean ± SD | Yield increase (%) | Mean ± SD | Yield increase (%) |
| Seed length (mm) | 6.41 ± 0.12 | 6.72 ± 0.12** | 4.81% | 6.73 ± 0.11** | 4.94% | 6.68 ± 0.14** | 4.16% |
| Seed width (mm) | 3.13 ± 0.07 | 3.28 ± 0.07* | 4.52% | 3.28 ± 0.09* | 4.79% | 3.28 ± 0.11* | 4.79% |
| Thousand seed weight (g) | 37.20 ± 1.53 | 40.84 ± 1.55** | 9.78% | 41.77 ± 2.40** | 12.27% | 41.53 ± 1.40** | 11.64% |
| Grain weight per plant (g) | 13.93 ± 2.20 | 14.93 ± 1.89 | 7.14% | 14.87 ± 2.50 | 6.72% | 14.96 ± 0.95 | 7.39% |
| Biomass (g) | 34.91 ± 6.78 | 36.16 ± 2.12 | 3.60% | 36.87 ± 4.63 | 5.61% | 37.38 ± 3.23 | 7.08% |
| Plant height (cm) | 112.73 ± 2.71 | 96.27 ± 2.11** | -14.60% | 92.18 ± 3.29** | -18.23% | 92.91 ± 1.38** | -17.58% |

The field trial was conducted under well-irrigated (WIR) conditions. A sowing density of 50 seeds per 2-meter row was used. For each replicate, the wild-type ‘Fielder’ and each transgenic overexpression line were planted in three adjacent rows. The experiment included three independent biological replicates. Three irrigations were applied during the growing season: at the pre-wintering, jointing, and grain-filling stages. Each irrigation delivered 750 m^3^ of water per hectare. Data are presented as mean ± standard deviation (SD) (n = 15, three biological replicates). Significant differences between transgenic overexpression lines and the wild-type ‘Fielder’ control were assessed using Student’s t-test (**P* < 0.05; ***P* < 0.01). The reported biomass refers to the fresh weight of the aboveground plant material. Abbreviations: SD, standard deviation; WIR, well-irrigated.

Table S5. Agronomic Performance of Wild-Type and *TaCOMT1A*-Overexpressing Wheat Under Limited-Irrigation Field Conditions (Shunyi, Beijing, 2022).

| **Traits** | **Fielder** | ***Ubi:TaCOMT1A#6*** | | ***Ubi:TaCOMT1A#8*** | | ***Ubi:TaCOMT1A#9*** | |
| --- | --- | --- | --- | --- | --- | --- | --- |
|  | Mean ± SD | Mean ± SD | Yield increase (%) | Mean ± SD | Yield increase (%) | Mean ± SD | Yield increase (%) |
| Seed length (mm) | 6.18 ± 0.07 | 6.65 ± 0.04** | 7.55% | 6.63 ± 0.11** | 7.21% | 6.63 ± 0.07** | 7.14% |
| Seed width (mm) | 2.97 ± 0.07 | 3.19 ± 0.06** | 7.58% | 3.18 ± 0.07** | 7.30% | 3.20 ± 0.06** | 7.87% |
| Thousand seed weight (g) | 34.45 ± 1.43 | 39.68 ± 2.31** | 15.17% | 40.42 ± 2.15** | 17.31% | 39.59 ± 1.28** | 14.89% |
| Grain weight per plant (g) | 8.89 ± 0.74 | 10.45 ± 1.22* | 17.50% | 10.52 ± 0.98* | 18.37% | 10.28 ± 1.17* | 15.58% |
| Biomass (g) | 17.21 ± 1.20 | 20.46 ± 1.83** | 18.87% | 20.49 ± 1.56** | 19.06% | 20.69 ± 2.74* | 20.23% |
| Plant height (cm) | 100.82 ± 3.62 | 90.09 ± 1.57** | -10.64% | 85.82 ± 1.89** | -14.88% | 86.91 ± 3.54** | -13.80% |
| Stress susceptibility index (SSI) | 1.13 ± 0.06 | 0.99 ± 0.10* | -12.39% | 0.92 ± 0.13** | -18.58% | 0.95 ± 0.14* | -15.93% |
| Stress tolerance index (STI) | 0.49 ± 0.06 | 0.59 ± 0.09** | 20.41% | 0.60 ± 0.05** | 22.45% | 0.60 ± 0.02** | 22.45% |
| Geometric mean productivity (GMP) | 13.23 ± 0.84 | 14.42 ± 1.08* | 8.99% | 14.67 ± 0.59** | 10.88% | 14.63 ± 0.30** | 10.58% |

The field trial was conducted under limited irrigation (LIR) conditions. Sowing density was standardized to 50 seeds per 2-meter row. For each replicate, the wild-type ‘Fielder’ and each transgenic overexpression line were planted in three adjacent rows. The experiment included three independent biological replicates. Two irrigations were applied during the growing season: at the pre-wintering stage and the jointing stage. Each irrigation delivered 750 m^3^ of water per hectare. Data are presented as mean ± standard deviation (SD) (n = 15, three biological replicates). Significant differences between transgenic overexpression lines and the wild-type ‘Fielder’ control were assessed using Student’s t-test (**P* < 0.05; ***P* < 0.01). The reported biomass refers to the fresh weight of the aboveground plant material. Abbreviations: SD, standard deviation; LIR, limited irrigation.

Table S6. Agronomic Performance of Wild-Type and *TaCOMT1A*-Overexpressing Wheat Under Well-Irrigated Field Conditions (Shunyi, Beijing, 2023).

| **Traits** | **Fielder** | ***Ubi:******TaCOMT1A#6*** | | ***Ubi:TaCOMT1A#8*** | | ***Ubi:TaCOMT1A#9*** | |
| --- | --- | --- | --- | --- | --- | --- | --- |
|  | Mean ± SD | Mean ± SD | Yield increase (%) | Mean ± SD | Yield increase (%) | Mean ± SD | Yield increase (%) |
| Seed length (mm) | 6.61 ± 0.31 | 6.95 ± 0.16** | 5.04% | 6.83 ± 0.16** | 3.28% | 6.84 ± 0.11** | 3.43% |
| Seed width (mm) | 3.04 ± 0.11 | 3.21 ± 0.11** | 5.43% | 3.22 ± 0.14** | 5.74% | 3.20 ± 0.08** | 5.37% |
| Thousand seed weight (g) | 44.15 ± 1.99 | 47.89 ± 2.71** | 8.46% | 48.86 ± 3.52** | 10.66% | 49.24 ± 2.96** | 11.51% |
| Grain weight per plant (g) | 18.49 ± 3.52 | 19.07 ± 3.79 | 3.15% | 18.92 ± 3.29 | 2.33% | 19.09 ± 4.67 | 3.24% |
| Biomass (g) | 36.60 ± 7.61 | 38.13 ± 7.10 | 4.17% | 38.07 ± 8.09 | 4.02% | 38.63 ± 8.80 | 5.54% |
| Plant height (cm) | 85.77 ± 2.88 | 75.34 ± 2.75** | -12.15% | 69.40 ± 3.42** | -19.08% | 70.49 ± 3.09** | -17.82% |

The field trial was conducted under well-irrigated (WIR) conditions. A sowing density of 50 seeds per 2-meter row was used. For each replicate, the wild-type ‘Fielder’ and each transgenic overexpression line were planted in three adjacent rows. The experiment included three independent biological replicates. Three irrigations were applied during the growing season: at the pre-wintering, jointing, and grain-filling stages. Each irrigation delivered 750 m^3^ of water per hectare. Data are presented as mean ± standard deviation (SD) (n = 15, three biological replicates). Significant differences between transgenic overexpression lines and the wild-type ‘Fielder’ control were assessed using Student’s t-test (**P* < 0.05; ***P* < 0.01). The reported biomass refers to the fresh weight of the aboveground plant material. Abbreviations: SD, standard deviation; WIR, well-irrigated.

Table S7. Agronomic Performance of Wild-Type and *TaCOMT1A*-Overexpressing Wheat Under Limited-Irrigation Field Conditions (Shunyi, Beijing, 2023).

| **Traits** | **Fielder** | ***Ubi:TaCOMT1A#6*** | | ***Ubi:TaCOMT1A#8*** | | ***Ubi:TaCOMT1A#9*** | |
| --- | --- | --- | --- | --- | --- | --- | --- |
|  | Mean ± SD | Mean ± SD | Yield increase (%) | Mean ± SD | Yield increase (%) | Mean ± SD | Yield increase (%) |
| Seed length (mm) | 6.28 ± 0.14 | 6.77 ± 0.17** | 7.80% | 6.70 ± 0.17** | 6.63% | 6.67 ± 0.18** | 6.26% |
| Seed width (mm) | 3.01 ± 0.12 | 3.30 ± 0.13** | 9.49% | 3.23 ± 0.12** | 7.24% | 3.27 ± 0.17** | 8.62% |
| Thousand seed weight (g) | 38.80 ± 3.02 | 43.99 ± 2.52** | 13.36% | 44.41 ± 2.50** | 14.46% | 44.76 ± 2.45** | 15.36% |
| Grain weight per plant (g) | 9.47 ± 2.10 | 10.83 ± 2.28* | 14.28% | 10.99 ± 2.10** | 16.04% | 10.84 ± 2.90* | 14.43% |
| Biomass (g) | 18.86 ± 4.67 | 21.60 ± 4.99* | 14.53% | 21.78 ± 5.60* | 15.47% | 21.24 ± 4.27* | 12.59% |
| Plant height (cm) | 71.35 ± 3.39 | 62.45 ± 4.12** | -12.47% | 63.56 ± 3.84** | -10.92% | 62.58 ± 4.00** | -12.29% |

The field trial was conducted under limited irrigation (LIR) conditions. Sowing density was standardized to 50 seeds per 2-meter row. For each replicate, the wild-type ‘Fielder’ and each transgenic overexpression line were planted in three adjacent rows. The experiment included three independent biological replicates. Two irrigations were applied during the growing season: at the pre-wintering stage and the jointing stage. Each irrigation delivered 750 m^3^ of water per hectare. Data are presented as mean ± standard deviation (SD) (n = 15, three biological replicates). Significant differences between transgenic overexpression lines and the wild-type ‘Fielder’ control were assessed using Student’s t-test (**P* < 0.05; ***P* < 0.01). The reported biomass refers to the fresh weight of the aboveground plant material. Abbreviations: SD, standard deviation; LIR, limited irrigation.

**Data S1.** Comparative flavonoid profiling of wild-type ‘Fielder’ and *TaCOMT1A*-overexpression line #9.

**Data S2.** RNA-Seq data summary for wheat cultivars ‘Shi4185’, ‘Chang6878’, and ‘Hanxuan10’.

**Data S3.** Differential expression genes analysis for wheat cultivar ‘Shi4185’.

**Data S4.** Differential expression genes analysis for wheat cultivar ‘Chang6878’.

**Data S5.** Differential expression genes analysis for wheat cultivar ‘Hanxuan10’.

**Data S6.** Sakuranetin-regulated drought response genes in wheat cultivars ‘Shi4185’, ‘Chang6878’, and ‘Hanxuan10’.
